# Discrimination methods for diesel origin by analyzing fatty acid methyl ester (FAME) composition in diesel-contaminated soil

**DOI:** 10.1038/s41598-021-95780-3

**Published:** 2021-08-10

**Authors:** Myoung-Soo Ko, Seunghak Lee

**Affiliations:** 1 Department of Energy and Resources Engineering, Kangwon National University, Chuncheon, 24341 Republic of Korea; 2 Department of Integrated Energy and Infra System, Graduate School, Kangwon National University, Chuncheon, 24341 Republic of Korea; 3 Water Cycle Research Center, Korea Institute of Science and Technology (KIST), Seoul, 02792 Republic of Korea

**Keywords:** Environmental impact, Sustainability, Environmental impact, Environmental sciences

## Abstract

The biodiesel containing fatty acid methyl esters (FAMEs) are blended with refined diesel products. Here, we evaluate relative FAME composition ratio as a potential index to discriminate the pollution origin in diesel-contaminated soil. Artificially contaminated soil was prepared to mimic the release of petroleum products using four different refined diesels; in addition, the contaminated soil was put under natural weathering conditions. The variations in the relative FAME composition ratio was compared with those of the corresponding diesel origin using principal component analysis (PCA) for 60 days. All soil samples could be classified into four groups according to diesel origin using two principal components. The proposed method can be used to discriminate the specific diesel pollution origin in contaminated soils.

## Introduction

Diesel is widely used as a commercial petroleum product; hence, it can be easily released into soil and water. Accidental exposure to various hydrocarbons such as fuel and refinery products, frequently occurs in the storage and transportation system. Diesel spill accounts for approximately 25% of annual spill accidents in Canada^1^. The identification of pollution origin is important to remediate diesel-contaminated media. A few studies have reported potential methods to identify the source of diesel contamination^2–5^. However, the discrimination of diesel refined by different refineries from the same crude oil remains a challenge.

In Korea, domestic diesel is produced from the same crude oil regardless of the refineries, and this limits the use of previously reported identification indices such as the diagnostic ratio of sesquiterpanes^5^ and polycyclic aromatic hydrocarbons (PAHs)^6^. Thus, there is a need for novel indices for diesel source identification. In this milieu, we exploited the fact that biodiesels have to be mandatorily blended with domestic diesel products (3% by volume) by the national policy. Generally, the refineries blend different biodiesels produced from empty fruit bunch and waste cooking oil. Fatty acid methyl esters (FAMEs) are the major compounds in biodiesels^7,8^. In this study, we estimated the FAME compositions in diesels as a potential index for discriminating specific diesel origin in contaminated soils. Four diesel products were collected from four major domestic refineries in Korea, and their relative FAME compositions were analyzed. Principal components analysis (PCA) was used to characterize the differences in the relative FAME composition in different diesel sources and soil samples artificially contaminated with each source. Furthermore, we estimated the weathering effect on the method using relative FAME composition.

## Materials and methods

### Materials

Four diesel products, blended with biodiesel, were obtained from gas stations operated by four major oil refineries in Korea, and the samples were designated as D1, D2, D3, and D4. The diesel samples were stored in amber glass bottles with screw caps at 4 °C. The FAME composition in each diesel sample was determined by dissolving an aliquot of diesel in dichloromethane (DCM, ≥ 99%, JT Baker), analyzing by gas chromatography-mass spectrometry (GC–MS, QP 2010, Shimadzu), and comparing with the FAME standard (FAMQ-005, AccuStandard). We collected soil in the hillside near the Korea Institute of Science and Technology (KIST); this region is not exposed to petroleum products. The distribution of soil particle size was determined by sieve separation. The abundant particle size range of the soil was 2–0.075 mm, which belongs to sand; in addition, approximately 25 wt% of soil particles were below 0.075 mm, indicating the presence of silt and clay particles. The collected soil was dried for 7 days at room temperature, passed through a 2 mm mesh size sieve, and then used to prepare diesel-contaminated soils^9^.

### Preparation of diesel-contaminated soils and extraction of FAME

The collected soil (2 kg) was thoroughly mixed with 200 g of diesel (10% by weight) to prepare diesel-contaminated soils. The apparent color of soil changed from light to dark brown after being mixed with diesel. The soils contaminated with different diesel products were compacted to a predetermined depth to achieve similar porosity. The diesel-contaminated soils were placed in opaque jars and stored under open condition in a fume hood during the experiment.

Soil samples were collected from the four different diesel-contaminated soils using a small hand auger with an inner diameter of 16 mm at 1, 30, and 60 days after being placed in the fume hood. To analyze FAMEs in the soil samples, DCM extraction was performed by modifying a previously reported procedure^10^. Briefly, 2 g of soil sample was mixed with 10 mL of DCM; the mixture was agitated on a rotary shaker at 40 rpm for 1 h, and the supernatant was analyzed by GC–MS after settling the suspension.

### Analytical method

FAMEs in the soil samples were analyzed by GC–MS equipped with an Omega Wax 250 column (Supelco, 30 m × 0.25 mm, thickness 0.25 µm)^11^ in the total ion chromatogram (TIC) and selective ion monitoring (SIM) modes. The oven temperature was programmed as reported previously^12,13^, and the details are summarized in Table 1.

**Table 1:** Analytical conditions of GC–MS.

Parameter	Conditions
Injector temperature	250 °C
Column oven temperature	180 °C
Split ratio	100
Column	Omega Wax 250 (30 m, 0.25 mm, 0.25 µm)
Oven temperature	180 °C (0 min)–4 °C/min–240 °C (5 min)
Carrier gas	He
Gas flow	86.9 mL/min
Pressure	85.6 kPa
Linear velocity	35 cm/s
Purge flow	3.0 mL/min

### Discrimination of specific diesel in soil samples using principal component analysis

**Figure 1:**
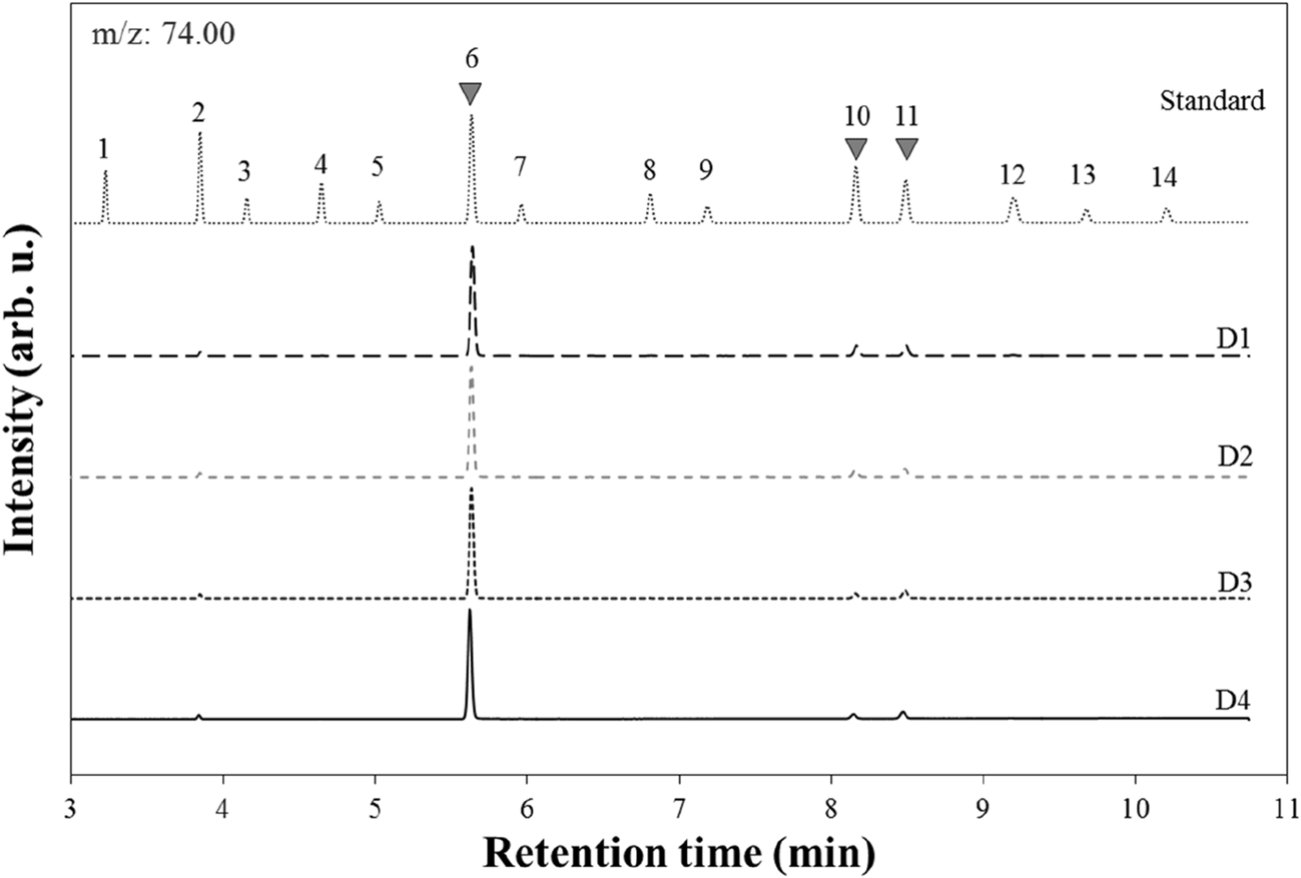
FAMEs in the standard and extracts of the diesel-contaminated soils analyzed by GC–MS in the SIM mode at m/z 74.00. Specific compounds for the peak numbers are listed in Table 2.

The FAMEs that were successfully detected in the extracts of diesel-contaminated soils were selected and used for discrimination. The relative FAME composition and proportion were evaluated for discrimination of the diesel sources in the contaminated soil. For the PCA, the relative proportion of the selected FAME was calculated using each peak area. The relative proportion of FAMEs was defined as the proportion of one FAME compound area to the total peak areas of the selected FAMEs. The difference in the relative FAME proportion in the four diesel samples was presented by two principal components (PC1 and PC2). The principal components of the diesel samples were calculated by the PCA with the following equations using SPSS (ver. 13.0)^14^.

1 $$ {\text{PC1}} = {\text{A}}_{{1}} {\text{X}}_{{1}} + {\text{A}}_{{2}} {\text{X}}_{{2}} + {\text{A}}_{{3}} {\text{X}}_{{3}} + \cdots + {\text{A}}_{{\text{n}}} {\text{X}}_{{\text{n}}} $$

2 $$ {\text{PC2}} = {\text{B}}_{{1}} {\text{X}}_{{1}} + {\text{B}}_{{2}} {\text{X}}_{{2}} + {\text{B}}_{{3}} {\text{X}}_{{3}} + \cdots + {\text{B}}_{{\text{n}}} {\text{X}}_{{\text{n}}} $$

where, A_n_ and B_n_ are the component scores obtained using the factor analysis in SPSS. X_n_ is calculated as (X − X_avg_)/X_stdev_, in which X represents the relative proportion of one FAME compound in diesel sample. Coordinates of soil extracts were calculated by component scores, X_avg_, and X_stdev_ derived from determining the coordinates of diesel sources. PC1 and PC2 could indicate the linear combination of each FAME component that produces the maximum difference among the four diesel groups. Thus, the calculated values can be used to allocate extractants of unknown samples to one of the four source groups. The diesel source in contaminated soils was discriminated by comparing the closeness of position of the extracts and the diesel sources in the PC1–PC2 plane (represented by Euclidean distance). Heptadecanoic acid methyl ester (HA), linolenic acid methyl ester (LA), and elaidic acid methyl ester (EA) were accurately measured at m/z 74 in the SIM mode (Fig. 1). Thus, the PCA was conducted for the relative proportion of HA, LA, and EA in the diesel sources and soil extracts to avoid the misjudgment driven by the disappearance of certain compounds while correlating the relative FAME compositions of the sources and samples.

## Results and discussion

### Identification of FAMEs in different diesel sources

FAMEs in each diesel product were identified by GC–MS in the TIC mode (Table 2). Different sets of FAMEs were detected in each diesel product, implying that the diesels used in this study might contain different kinds of biodiesels. D1 and D2 showed additional peaks for cis-5,8,11,14,17-Eicosapentaenoic acid methyl ester whereas cis-11-14-17-Eicosatrienoic acid methyl ester was detected only in D2. The HA, LA, and EA were commonly found in all diesel products; hence, these three FAME compounds were used to discriminate the diesel sources in the contaminated soil.

**Table 2:** List of FAMEs in the standard and diesel sources analyzed in the TIC mode. ■ indicate that the FAME was detected and □ implies not detected in each diesel sample.

Peak no.	FAME (CAS no.)	Retention time (min)	D1	D2	D3	D4
1	Tridecanoic acid methyl ester (1731-88-0)	3.24	□	□	□	□
2	Myristoleic acid methyl ester (56219-06-8)	3.86	□	□	□	□
3	Pentadecanoic acid methyl ester (7132-64-1)	4.17	□	□	□	□
4	Palmitic acid methyl ester (112-39-0)	4.66	□	□	□	□
5	cis-10-Heptadecenoic acid methyl ester (75190-82-8)	5.04	□	□	□	□
6	Heptadecanoic acid methyl ester (1731-92-6)	5.65	■	■	■	■
7	Linolelaidic acid methyl ester (2566-97-4)	5.98	□	□	□	□
8	Oleic acid methyl ester (112-62-9)	6.83	□	□	□	□
9	Linoleic acid methyl ester (112-63-0)	7.20	□	□	□	□
10	Linolenic acid methyl ester (301-00-8)	8.18	■	■	■	■
11	Elaidic acid methyl ester (1937-62-8)	8.51	■	■	■	■
12	Stearic acid methyl ester (112-61-8)	9.22	□	□	□	□
13	Arachidonic acid methyl ester (2566-89-4)	9.70	□	□	□	□
14	cis-5,8,11,14,17-Eicosapentaenoic acid methyl ester (2734-47-6)	10.23	■	■	□	□
15	cis-11-14-17-Eicosatrienoic acid methyl ester (55682-88-7)	11.28	□	■	□	□
16	Arachidic acid methyl ester (1120-28-1)	11.64	□	□	□	□
17	cis-13,16-Docosadienoic acid methyl ester (61012-47-3)	12.46	□	□	□	□
18	Behenic acid methyl ester (929-77-1)	12.95	□	□	□	□

### Discrimination of diesel origin in soils by analyzing the relative FAME composition

**Figure 2:**
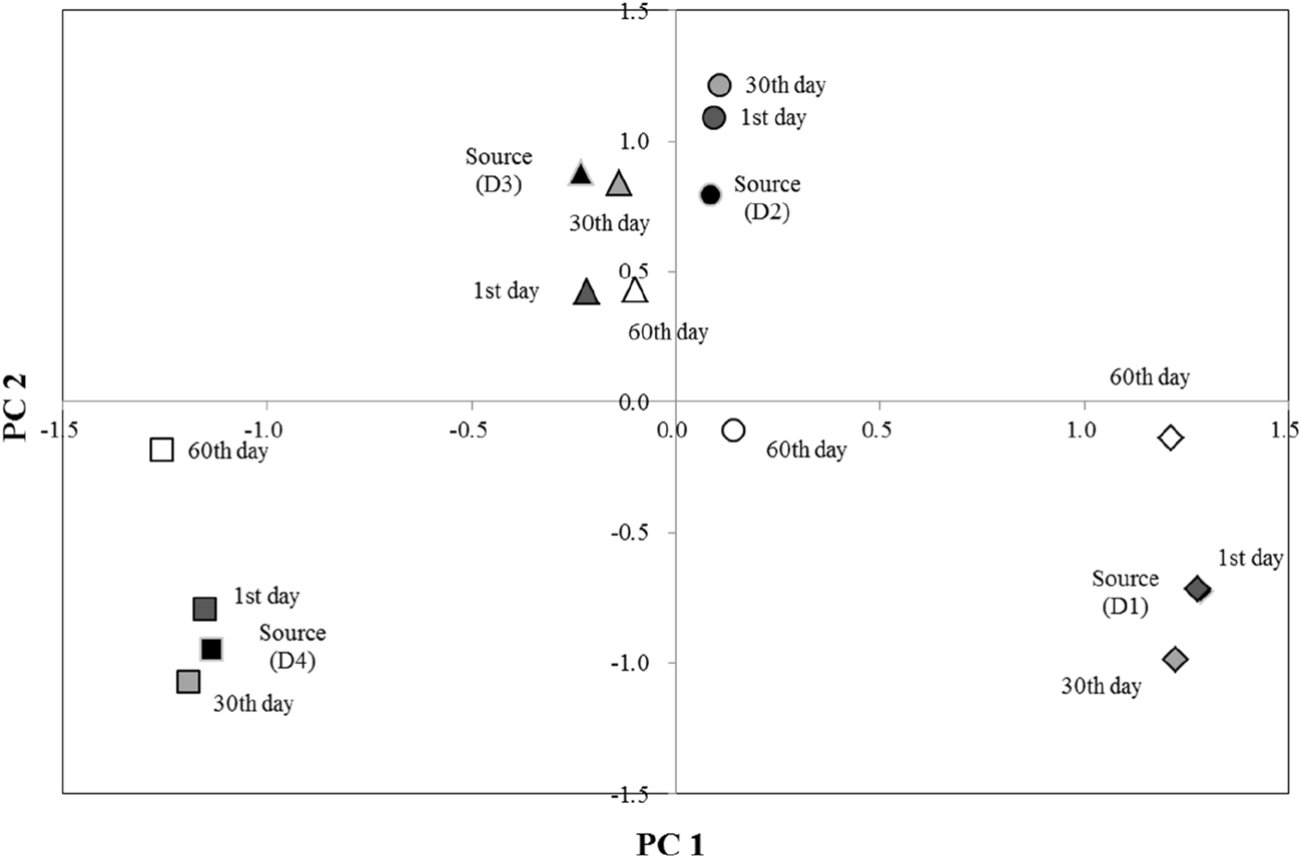
PCA for the relative FAME compositions and proportion in the diesel sources and soil samples contaminated with the corresponding sources at different weathering time; ♦ D1, ● D2, ▲ D3, and ■ D4.

DCM extract of soil not exposed to diesel did not show distinguishable peaks for FAMEs on GC–MS chromatograms, indicating that the interference by natural compounds in soils on the analysis is negligible. Although the diesel sources presented at least four common FAME compounds, only three compounds were successfully detected in the extracts of diesel-contaminated soils, namely HA, LA, and EA. Relative FAME composition in the diesel sources and extracts of soil samples is summarized in Table 3. The HA was the most dominant compound with the relative composition of 78.5% ± 3.4%, whereas the composition of LA and EA was 6.8% ± 1.8% and 14.8% ± 1.7%, respectively. The PCA discriminated the diesel sources based on the differences in the relative FAME composition. All diesel samples were distributed at different locations in the PC1–PC2 plane (Fig. 2): D1 in the 4th quadrant, D2 in the 1st quadrant, D3 in the 2nd quadrant, and D4 in the 3rd quadrant. The cumulative eigenvalue of the two principal components was 99.97%. The soil extracts at different degrees of weathering were plotted in the PC1–PC2 plane. Each soil sample point, except that for day 60, gathered around their source diesel. The PCA results imply that the relative FAME composition might be applied as an index to discriminate diesel origin in the contaminated soil.

**Table 3:** Relative FAME compositions in the diesel sources and soil samples contaminated with the corresponding sources at different weathering times.

Unit: %												
FAME components	D1			D2			D3			D4		
	HA^a^	LA^b^	EA^c^	HA	LA	EA	HA	LA	EA	HA	LA	EA
Source	74.1	8.7	17.2	78.2	7.2	14.7	79.2	6.7	14.1	82.3	4.4	13.3
Day 1	73.9	8.8	17.3	77.9	7.4	14.7	79.0	6.6	14.5	82.2	4.5	13.3
Day 30	78.8	9.2	12.0	81.9	7.9	10.2	82.6	7.3	10.1	86.0	4.6	9.4
Day 60	82.0	9.9	8.1	85.4	7.7	6.9	86.1	7.5	6.5	89.8	4.8	5.4

^a^HA: Heptadecanoic acid methyl ester.

^b^LA: Linolenic acid methyl ester.

^c^EA: Elaidic acid methyl ester.

### Weathering effect on the discrimination method using relative FAME compositions

**Figure 3:**
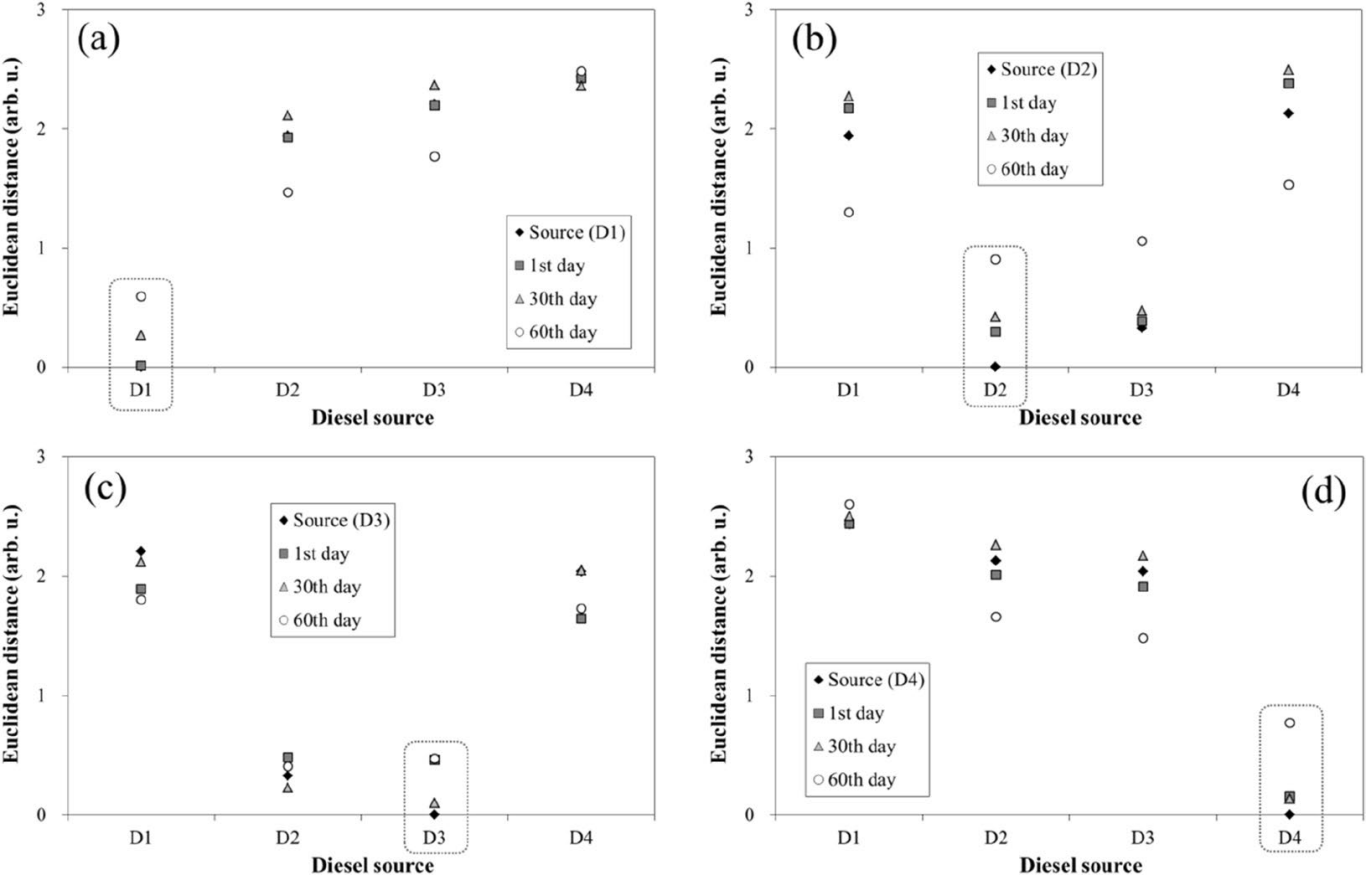
Euclidean distance between data points of the diesel sources and soil samples contaminated with (**a**) D1, (**b**) D2, (**c**) D3, and (**d**) D4.

Euclidean distances from a sample point to all of the diesel source points in the PC1–PC2 plane are presented in Fig. 3. In general, all samples showed the shortest Euclidean distance to the corresponding diesel source. However, the Euclidean distance between an extract from soil sample and the corresponding source increased with time. This can be attributed to different weathering resistances of each FAME in diesel^15,16^. The weathering effect implies that the diesel source discrimination method using relative FAME composition is subject to a degree of error, especially in the case of highly weathered samples.

## Conclusions

Biodiesels are considered an alternative to conventional refined petroleum and are widely used in several countries; they are also used in biodiesel-blended diesel. Relative FAME composition and proportion of biodiesel and biodiesel-blended diesel might be employed as an index to discriminate the diesel source in diesel-contaminated soil. However, environmental weathering might affect the accuracy of the proposed discrimination method. To verify the applicability of this method, further studies should focus on the long-term variations in FAME compositions in diesel under more environmentally relevant conditions using various biodiesel-blended products.
